# From blood vessels to lymphatics and back again

**DOI:** 10.1152/function.102.2025

**Published:** 2025-12-08

**Authors:** Walter L. Murfee, Jerome W. Breslin

**Affiliations:** ^1^J. Crayton Pruitt Family Department of Biomedical Engineering, University of Florida, Gainesville, Florida, United States; ^2^Department of Molecular Pharmacology & Physiology, University of South Florida, Tampa, Florida, United States

From blood vessels to lymphatics and back again—the path for fluid transport from the circulation, through the interstitial space, into lymphatic capillaries, and eventually back into large veins, in many ways parallels the impacts made by researchers during our careers. Recognizing the overlaps between both systems, leading vascular physiologists leveraged opportunities to apply methods, approaches, and a fundamental understanding to elucidate how lymphatic vessels work (1, 2). In this journal, Davis and King (3) provide a nice example of in turn applying what they have learned about lymphatic function to advance our understanding of how blood vessels work (Fig. 1). Based on servo-nulling micropipette pressure measurements in isolated veins from mice, the results implicate a causal relationship between venous tone and venous valve closure.

**Figure 1.**
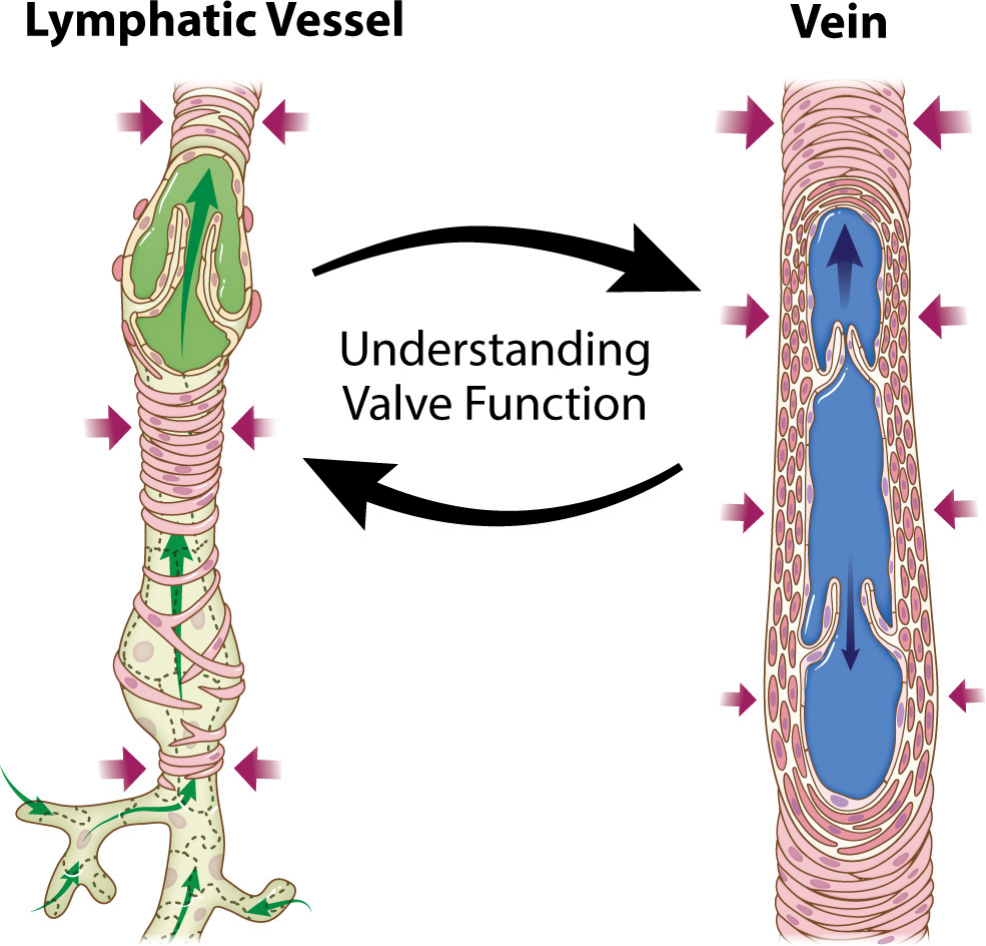
Application of our lymphatic biology understanding to advance our understanding of how venous valves work. The study by Davis and King (3) extends technically challenging measurements of pressures within isolated vessels and endothelial specific reporter mice used to characterize secondary lymphatic valve structure and function to suggest an underappreciated mechanism relating loss of venous tone and impaired venous valve closure. The study motivates the conceptual advance in the context of what can be learned by recognizing the overlaps between lymphatic and vascular research. Printed with permission from Anita lmpagliazzo © 2025.

The work refreshingly extends methods used to describe pressure differences across valves in isolated lymphangions (4, 5) to link the contractile state of venous smooth muscle cells to venous valve leak. Scientific rigor is highlighted by a simple story based on technically challenging physiology methods, which are less common these days, probably due to the length of required training time and the juxtaposition with pace-driven pressures of academic research. The application of the old methods provokes new questions and reconsideration of what we know about venous smooth muscle cell constriction and venous valve function. How many valves are needed along veins? Are venous valves heterogenous? Does heterogeneity matter? Can rescuing venous tone improve venous valve function? Can the relationship between venous tone and valve function explain dysfunction associated with disease states? As Dr. Davis passes his servonulling systems to the next generation of vascular physiologists, maybe the most pressing question is—who will be measuring pressures?

## References

[1] Breslin JW, Yang Y, Scallan JP, Sweat RS, Adderley SP, Murfee WL. Lymphatic vessel network structure and physiology. Compr Physiol 9: 207–299, 2018. doi:10.1002/cphy.c180015. 30549020 PMC6459625

[2] Lampejo AO, Jo M, Murfee WL, Breslin JW. The microvascular-lymphatic interface and tissue homeostasis: critical questions that challenge current understanding. J Vasc Res 59: 327–342, 2022. doi:10.1159/000525787. 36315992 PMC9780194

[3] Davis MJ, King PD. Venous tone is a critical determinant of venous valve closure in the mouse. Function (Oxf) 6: zqaf052, 2025. doi:10.1093/function/zqaf052. 41183494 PMC12658363

[4] Davis MJ, Castorena-Gonzalez JA, Li M, Simon AM, Srinivasan RS. Hierarchical requirement for endothelial cell connexins Cx37, Cx47, Cx43 and Cx45 in lymphatic valve function. Function (Oxf) 6: zqaf034, 2025. doi:10.1093/function/zqaf034. 40720769 PMC12448487

[5] Davis MJ, Zawieja SD, Yang Y. Developmental progression of lymphatic valve morphology and function. Front Cell Dev Biol 12: 1331291, 2024. doi:10.3389/fcell.2024.1331291. 38450249 PMC10915029

